# Bolus Versus Continuous Enteral Feeding for Critically Ill Patients: A Systematic Review and Meta-Analysis

**DOI:** 10.7759/cureus.54136

**Published:** 2024-02-13

**Authors:** Ahmed M Abdelbaky, Wael G Elmasry, Ahmed H. Awad

**Affiliations:** 1 Intensive Care Unit, Rashid Hospital - Dubai Health, Dubai, ARE

**Keywords:** bolus enteral feeding, continuous enteral feeding, critically ill patients, icu, enteral feeding

## Abstract

Enteral feeding is a crucial aspect of nutritional support for critically ill patients. However, the optimal feeding approach, whether bolus or continuous, remains a subject of debate. This systematic review and meta-analysis aimed to compare the outcomes of bolus feeding and continuous enteral feeding in critically ill patients. A systemic search was carried out in PubMed, Cumulative Index to Nursing and Allied Health Literature (CINAHL) Ultimate, Web of Science, Scopus, and Google Scholar to identify relevant studies. To ensure that we obtain the latest evidence on the topic, the search was limited to the last five years. Risk of bias assessments and meta-analyses were performed for relevant clinical outcomes. A total of nine randomized controlled trials (RCTs) were included, involving a total of 863 patients. All the studies were published between 2020 and 2023. High-risk performance bias was observed in seven studies, with unclear risk in two studies. In terms of clinical outcomes, no statistically significant differences were found between bolus and continuous enteral feeding in terms of diarrhea (odds ratio {OR} 0.60, 95% CI 0.27 to 1.30, p=0.20), constipation (OR 1.52, 95% CI 0.91 to 2.53, p=0.11), vomiting (OR 0.74, 95% CI 0.36 to 1.49, p=0.39), distention (OR 0.70, 95% CI 0.14 to 3.58, p=0.66), aspiration (OR 0.61, 95% CI 0.16 to 2.73, p=0.48), and gastric residual volume (GRV) (OR 0.80, 95% CI 0.30 to 2.15, p=0.66). Furthermore, no significant differences between bolus and continuous feeding were observed in terms of intensive care unit (ICU) mortality (OR 0.66, 95% CI 0.42 to 1.04, p=0.07), hospital mortality (OR 0.57, 95% CI 0.31 to 1.03, p=0.06), ICU length of stay (OR 0.70, 95% CI 0.50 to 1.90, p=0.25), and hospital length of stay (OR -0.86, 95% CI -3.04 to 1.33, p=0.44). This systematic review and meta-analysis suggest that bolus and continuous enteral feeding methods exhibit comparable outcomes in critically ill patients. However, both ICU mortality and hospital mortality outcomes were close to achieving statistical significance, which favored the continuous feeding approach.

## Introduction and background

The intensive care unit (ICU) is a fundamental section of any medical facility where critically ill patients receive treatments. In such patients, the provision of nutritional support is a foremost necessity. Satisfactory nutritional therapy can enhance the medical outcomes, nutritional status, and immune defense mainly associated with malnutrition [1]. Prior studies have demonstrated that adequate nutritional provision can improve patient outcomes and survival [2,3]. Some estimates suggest that approximately 10-60% of patients admitted in hospital are malnourished which can have serious implications for such patients [4]. Furthermore, critical illness has associations with catabolic stress which enhances the multiorgan dysfunction risk, prolonged interval of stay in the medical center, and increased mortality and morbidity [5]. Early <48 hours of progressive nutrition supplement initiation in critically ill persons with suitable protein provision might reduce the catabolism, and improve integrity of the gastrointestinal tract [6]. Enteral nutrition (EN) is also a preferred nutritional source for infants in neonatal intensive care units [7].

Parental and EN have been widely described as methods of nutritional support; however, EN has several advantages over parental nutritional support. EN is more cost-effective and safer and it does not require central venous line access. EN aims to deliver nutrients to enhance the functioning of the immune system and prevent bacterial dislocation by maintaining gastrointestinal tract integrity, reducing muscle catabolism, and optimizing mucosal host defense [8]. On the other hand, critically ill patients are also affected by gastrointestinal impairment due to some factors including gastric stasis, postoperative ileus, gut hypofusion, and administration of some sedatives and certain antibiotics [9]. Despite the familiarity and widespread use of EN, the ideal dosing method is still controversial. Therefore, there is a need to further search for the optimal delivery method for EN. There are two main approaches to EN administration, intermittent/bolus and continuous EN. Intermittent bolus feeding involves administering EN multiple times a day, typically between four and six sessions, using a syringe, gravity pump, or feeding pump, with a dose of 250-750 mL. In contrast, continuous feeding employs a steady, hourly delivery over a 24-hour period through an electric feeding pump [10].

In the ICU, EN is commonly provided at a continuous degree that is consistent with the recent guidelines [11]. It is thought that continuous feeding might be linked with a lower delivery of nutrition as compared to intermittent boluses where nutritional administration needs termination to asses for extubation or to facilitate the investigation. It can also confine patient movement and can alter the hormonal secretion of intestines leading to long-term metabolic complexities including insulin resistance and hyperglycemia [8]. Intermittent EN is considered a more physiological approach as it enhances protein synthesis and mobility, and aids in maintaining the digestion and secretion of gastrointestinal hormones [10]. On the other hand, clinical studies have indicated that intermittent EN has enhanced the risk of feeding intolerance in ICU patients [9,11]. Other concerns related to intermittent EN administration are increased diarrhea and the possible risks of aspiration [9]. In a recent study, McNelly et al. described that intermittent nutrition was less disordered by diarrhea and vomiting and it provided superior nutritional delivery in contrast to continuous feeding in patients with serious illness [12].

The findings of the studies mentioned above present a complex and conflicting picture for medical practitioners, leaving uncertainty about the preferable EN strategy for critically ill patients. Moreover, the safety of the two EN strategies is still controversial. Taken together both continuous and intermittent administrations might be favored depending on the clinical situation. Previously, some systemic reviews and meta-analyses tried to analyze bolus versus continuous enteral feeding; however, they did not include recent data [8,10]. Therefore, we decided to perform a meta-analysis of randomized controlled trials (RCTs) published in the last five years.

## Review

Methodology

The present meta-analysis was conducted according to the published guidelines of the Cochrane Handbook for Systematic Reviews of Interventions [13]. The predetermined protocol was duly registered in the International Prospective Register of Systematic Reviews (PROSPERO) with the registration number CRD42024497668. Furthermore, this systemic review and meta-analysis also adhered to the Preferred Reporting Items for Systematic Reviews and Meta-Analyses (PRISMA) guidelines [14]. This meta-analysis only focused on RCTs, published in the last five years. As all the included studies already received ethical approval before publication, there was no need for ethical approval for this meta-analysis. The PICO framework for this systemic review and meta-analysis was as follows: patients (P) critically ill patients; intervention (I) bolus versus continuous enteral feeding; control (C) bolus versus continuous enteral feeding; and outcomes (O) incidence of diarrhea, constipation, vomiting, distention, aspiration, gastric residual volume (GRV), ICU mortality, hospital mortality, ICU length of stay, and hospital length of stay.

Search Strategy and Data Sources

Several key databases were explored to identify the relevant studies on bolus versus continuous enteral feeding for critically ill patients. The searched databases included PubMed, Cumulative Index to Nursing and Allied Health Literature (CINAHL) Ultimate, Web of Science, and Scopus. The search strategy included the use of keywords that were joined with Boolean operators “OR” and “AND.” The keywords used included “intensive care units,” “critical care,” “continuous,” “bolus,” “feeding,” and “enteral feeding.” The detailed search strategy used for this meta-analysis is presented in the appendix. To further expand the literature search, Google Scholar was also explored. The inclusion criteria defined for this meta-analysis included - (1) studies that compared patient outcomes in bolus versus continuous enteral feeding, (2) studies that focused on critically ill patients, and (3) studies that were published in the English language. The exclusion criteria included studies that did not compare bolus and continuous feeding regimens and did not focus on critically ill patients. To ensure that the latest evidence was summarized in this meta-analysis, the search was restricted to the last five years only. RCTs published before that were not considered.

Data Extraction

After retrieving results from the database search, the files were transferred to a reference manager for combining all the search results. The reference manager used in this meta-analysis was EndNote. At this stage, any duplicate records were removed. After the removal of duplicates, a combined research file was then uploaded to Rayyan (Cambridge, MA: Rayyan Systems, Inc.), a software specifically built for conducting systematic reviews [15]. At the crucial stage of study selection, two independent reviewers were actively involved in this process. For unbiased decision-making, reviewers had activated "blind" in Rayyan so they would not see each other’s selections. Firstly, judgments were made based on record titles and abstracts. In the second stage, the blind was removed and inclusion or exclusion decisions were compared until a consensus resulted. In cases of conflict, a third reviewer was approached to give their input and help in the final decision-making process. Finally, all relevant information regarding the studies was extracted in an Excel file including demographic properties and study outcomes.

Risk of Bias Assessment

For the risk of bias assessment, two authors were involved. The risk of bias was detected by Review Manager (RevMan) version 5.3 software (London, UK: Cochrane Library). The evaluation considered various crucial factors, including selection bias, concealment of allocation, performance bias, detection bias, attrition bias, reporting bias, and other biases. In case of any conflicts, a third author was involved.

Outcomes Measures

The primary outcomes were ICU mortality, hospital mortality, ICU length of stay, and hospital length of stay. Secondary outcomes included incidence of diarrhea, constipation, vomiting, distention, aspiration, and gastric residual volume (GRV).

Data Analysis

The present meta-analysis employed the DerSimonian and Laird method, incorporating a random-effects model to address potential heterogeneity among studies [16]. This method yields a consolidated estimate of the effect size, represented by the odds ratio (OR) for binary outcomes, accompanied by a 95% confidence interval (CI). The heterogeneity among the studies was evaluated using the I² statistic, which measures the proportion of total variation attributable to heterogeneity rather than random chance. A value of I² greater than 50% suggests considerable heterogeneity. The findings of the meta-analysis are depicted through forest plots, displaying combined effect sizes along with their associated 95% CIs. Statistical significance was determined by p-values, with a cutoff set at 0.05.

Results

Included Studies

**Figure 1:**
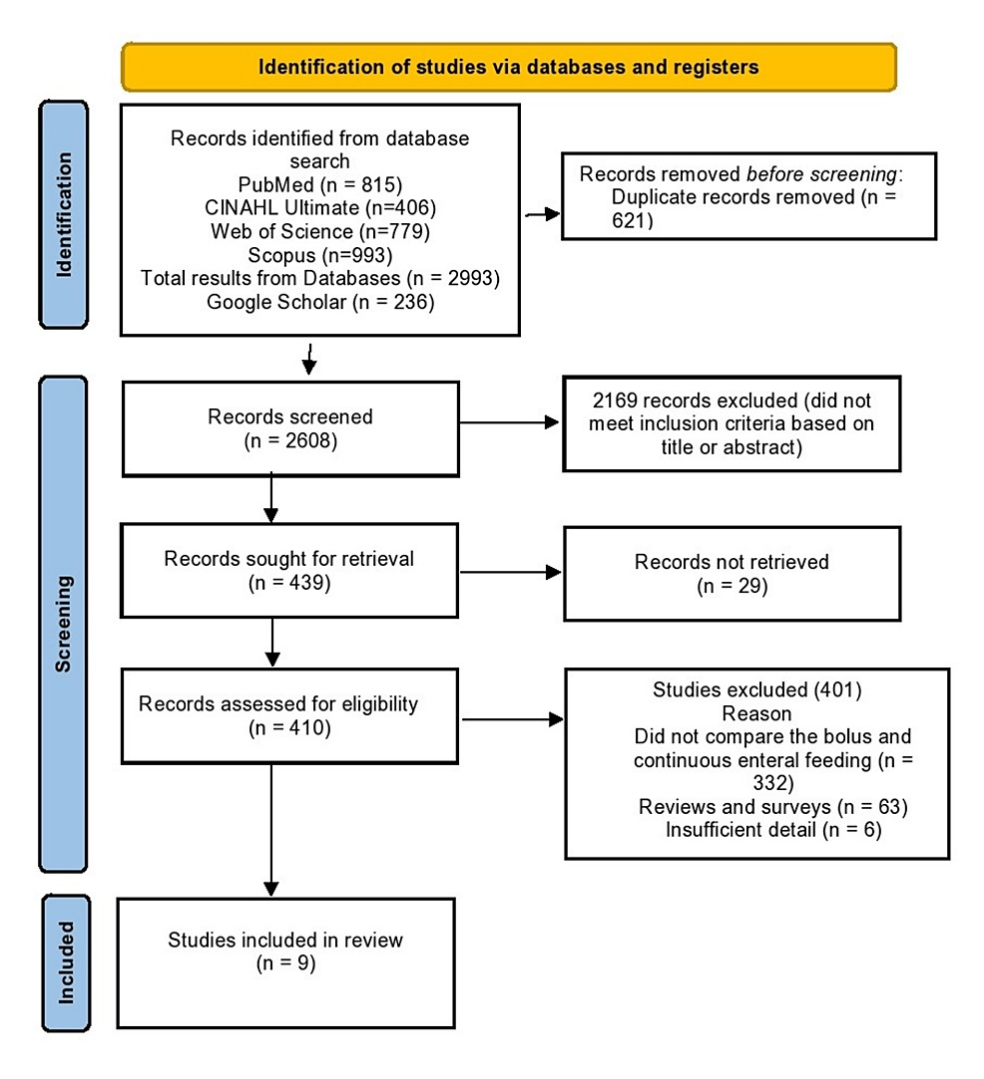
PRISMA flow diagram of the systemic review and meta-analysis. CINAHL: Cumulative Index to Nursing and Allied Health Literature; PRISMA: Preferred Reporting Items for Systematic Reviews and Meta-Analyses

The systemic search conducted in various databases revealed 2993 studies (PubMed=815, CINAHL Ultimate=406, Web of Science=779, and Scopus=993). Additionally, 236 studies were identified from Google Scholar. After removing duplicated studies, 2608 were finalized for screening. Of the screened records, 2169 articles did not meet the inclusion criteria based on title and abstract. Finally, after removing all non-relevant articles, nine RCTs were finalized for meta-analysis. Figure 1 shows the PRISMA flow diagram.

Characteristics of Included Studies

A total of 863 participants were included in nine RCTs. The ratio of bolus and continuous feeding arm was 1:1, with 430 patients included in the continuous feeding group and 433 in the bolus feeding group. The descriptive details of all the included studies are presented in Table 1.

**Table 1:** A brief description of the included trials. ICU: intensive care unit; LOS: length of stay; GRV: gastric residual volume; N/A: not attempted

Author and year	Selected population	No. of patients bolus feeding/ continuous feeding	Study arm		Major findings	Outcomes
			Bolus	Continuous		
Wilkinson et al. (2023) [17]	Aged ≥18 years critically ill patients	62/59	Six feeds per 24 h	Continuously over 24 h	Differences in metabolite patterns across time were not statistically significant between both groups	Intermittent and continuous feeding arms are comparable regarding changes in metabolic patterns
Veldscholte et al. (2023) [18]	Critically ill children below 18 years, children who were staying in PICU ≥48 h	67/73	Overnight fast	24 h	Condensed feeding periods during the day do not lead to more feeding intolerance	Intermittent feeding with an overnight fast resulted in lower calorie intake. Severe adverse events were the same in both groups
Banaei et al. (2022) [4]	Patients of trauma admitted to the ICU	36/36	N/A	N/A	There was no difference in both modalities concerning respiratory aspiration, intestinal excretion, and vomiting (p< 0.05)	Continuous feeding is a suitable method used in ICU to support the feeding method as GRV did not increase
Lee et al. (2022) [19]	Patients who require mechanical ventilation and who are ≥20 years of age	49/50	4 times a day for 1 h and 150 mL/h was the initial rate	24 h per day and 25 mL/h was the initial rate	Targeted nutrition was achieved ≥80% more significantly in the continuous group (65% versus 52.4%, p=0.008)	No significant difference was found in terms of clinical outcomes between the two feeding modalities including ICU LOS, gastrointestinal intolerance, mortality, and organ support
Ren et al. (2021) [20]	Patients who are admitted to the ICU	32/30	3 times for 2 h per day	24 h per day	Physiological and demographic differences between the two groups were not significant (p>0.050) No significant variance was found in the nutritional intolerance throughout the 7 days (p>0.050)	Intermittent feeding and continuous feeding have similar safety
Seyyedi et al. (2020) [21]	Patients who need mechanical ventilation in the ICU of Imam Khomeini Hospital and belong to the 18 to 85 years age group	17/17	75cc after 3 h	25cc per h	There was no significant difference in the serum phosphorus level between the control and intervention groups (p=0.22) and (p=0.14) after one week	Both groups increased the serum phosphorus level and indicated that for the control of phosphorus levels in critically ill patients nutritional support is important. On the other hand, there was no difference in blood glucose control in both groups
Zhu et al. (2020) [22]	Patients diagnosed with hemorrhagic strokes, ≤12 on GCS, and ≥18 years of age	40/38	4 times per day for 0.5 to 1 h	24 h per day with a maximum rate of 100 mL/h	The total intolerance rate and diarrhea were lower in the continuous group (p=0.027 and p=0.002, respectively)	There was no evidence to support the benefits of calorie intake in continuous tube feeding
Rana et al. (2021) [23]	Patients who are above 20 years, critically ill, and admitted to the ICU of Swami Rama Himalayan	68/68	150 to 250 eternal nutrition over a certain period of time	20 to 50 mL/h feeding with advancement by 10 to 25 mL every single 4 to 24 h	No incidence was found regarding tube displacement, gastric aspiration, tube obstructions, and vomiting. Gastric aspiration was seen on 2nd day, diarrhea (6.7%) was seen in the bolus group while in the continuous group diarrhea incidence was 3.3%. Except for co-morbidity, there was no statistical difference in other clinical outcomes (p>0.05)	Bolus and continuous feeding methods had similar outcomes in terms of complications. Hence, it remains controversial that eternal nutrition has fewer complications
McNelly et al. (2020) [12]	Patients who require mechanical ventilation for 48 h with ≥18 years of age	62/59	After every 4 h 1 bolus and a total of 6 bolus	24 h per day total volume feed	Muscle loss was the same between the two arms (p=0.0676). The safety profiles, discharge destination, physical function milestones, and gastric intolerance differences were not statistically significant between the two groups	In severely ill patients intermittent nutritional feeding revealed no muscle mass despite achieving greater nutritious goals than continuous feeding. It is a safe and feasible method

Risk of Bias

**Figure 2:**
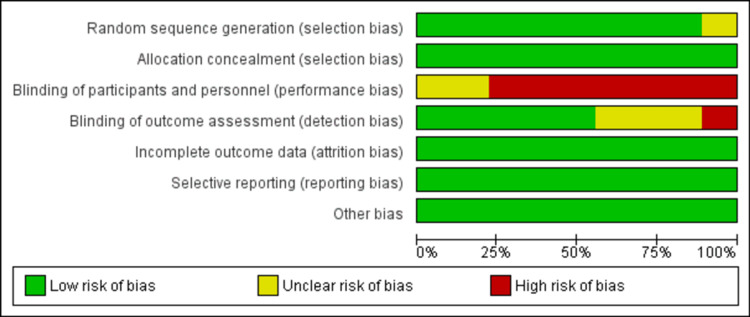
Risk of bias graph.

**Figure 3:**
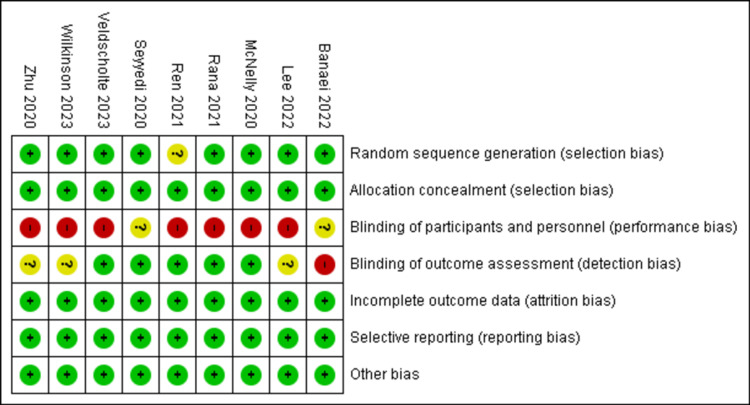
Risk of bias summary.

Figure 2 and Figure 3 show the risk of bias assessment of all the RCTs. A minimal risk of bias in random sequence creation was identified in eight out of nine papers (89%). All studies (100%) demonstrated a minimal risk of selection bias, attrition bias, reporting bias, and other biases. Performance bias was deemed high in seven studies, while two studies had an unclear risk of bias in this aspect. Only one study exhibited a high risk of bias in blinding of outcome assessment [4,12,17-23].

Primary Outcomes

**Figure 4:**
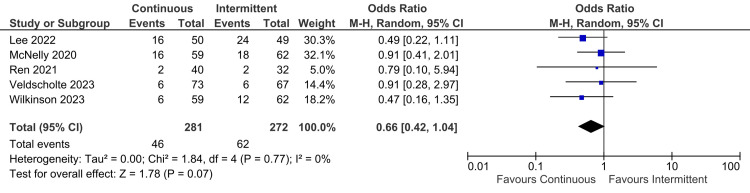
Forest plot representing ICU mortality. SD: standard deviation; CI: confidence interval; MH: Mantel-Haenszel; ICU: intensive care unit

ICU mortality: Only five out of nine studies reported ICU mortality outcomes. No statistically significant difference was observed in continuous and intermittent enteral feeding groups (OR: 0.66; 95% CI: 0.42 to 1.04; p=0.07; I^2^=0% {low heterogeneity}) (Figure 4) [12,17-20].

**Figure 5:**
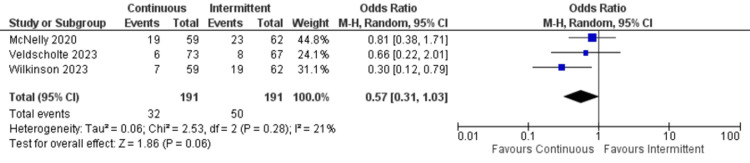
Forest plot of in-hospital mortality. CI: confidence interval; MH: Mantel-Haenszel

Hospital mortality: Only three studies reported hospital mortality. Similarly, there was no significant difference between continuous and intermittent enteral feeding groups (OR: 0.57; 95% CI: 0.31 to 1.03; p=0.06; I^2^=21% {low heterogeneity}) (Figure 5) [12,17,18].

**Figure 6:**
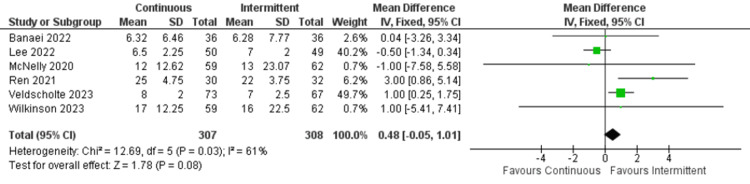
Forest plot of ICU length of stay. SD: standard deviation; CI: confidence interval; M-H: Mantel-Haenszel; ICU: intensive care unit

ICU length of stay: Regarding ICU length of stay, only three studies reported this outcome. Both continuous and intermittent enteral feeding groups demonstrated no significant differences (OR: 0.48; 95% CI: -0.05 to 1.01; p=0.08). However, the heterogeneity was high (I^2^=61%) (Figure 6) [4,12,17-20].

**Figure 7:**
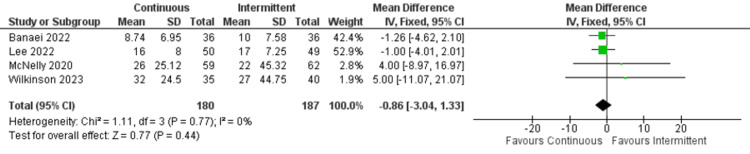
Forest plot of hospital length of stay. SD: standard deviation; CI: confidence interval

Hospital length of stay: Data from three studies was analyzed for hospital length of stay. Similarly, there was no significant difference between continuous and intermittent enteral feeding (OR: -0.86; 95% CI: -3.04 to 1.33; p=0.44; I^2^=0% {low heterogeneity}) (Figure 7) [4,12,17,19].

Secondary Outcomes

**Figure 8:**
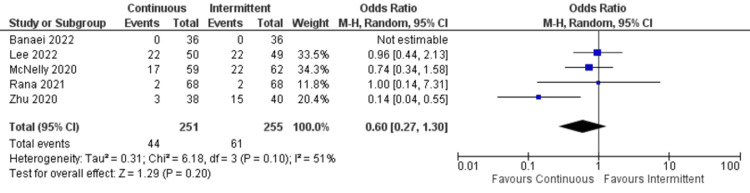
Forest plot representing incidence of diarrhea. CI: confidence interval; M-H: Mantel-Haenszel

Diarrhea: Only five studies reported data about constipation. There was no significant difference between continuous and intermittent enteral feeding (OR: 0.60; 95% CI: 0.27 to 1.30; p=0.20; I^2^=51% {moderate heterogeneity}) (Figure 8) [4,12,19,22,23].

**Figure 9:**
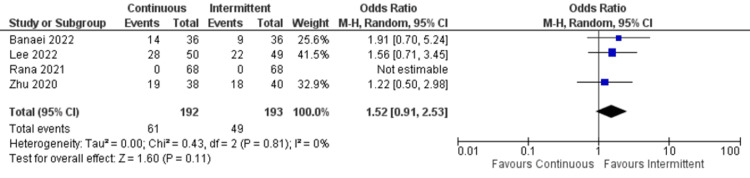
Forest plot representing incidence of constipation. CI: confidence interval; M-H: Mantel-Haenszel

Constipation: Only four studies reported data about constipation. There was no significant difference between continuous and intermittent enteral feeding (OR: 1.52; 95% CI: 0.91 to 2.53; p=0.11). There was no heterogeneity (I^2^=0%) in the studies (Figure 9) [4,19,22,23].

**Figure 10:**
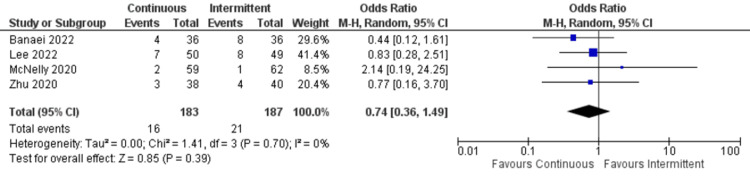
Forest plot of vomiting outcome. CI: confidence interval; M-H: Mantel-Haenszel

Vomiting: Four studies reported data for vomiting. Both continuous and intermittent enteral feeding arms showed no statistically significant differences (OR: 0.74; 95% CI: 0.36 to 1.49; p=0.39; I^2^=0% {low heterogeneity}) (Figure 10) [4,19,12,22].

**Figure 11:**
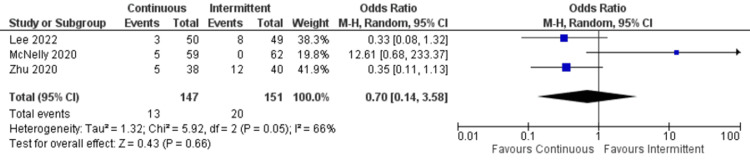
Forest plot of distention. CI: confidence interval; M-H: Mantel-Haenszel

Distention: Only three studies reported data for distention. There was no significant difference between continuous and intermittent enteral feeding (OR: 0.70; 95% CI: 0.14 to 3.58; p=0.66; I^2^=66% {high heterogeneity}) (Figure 11) [12,19,22].

**Figure 12:**
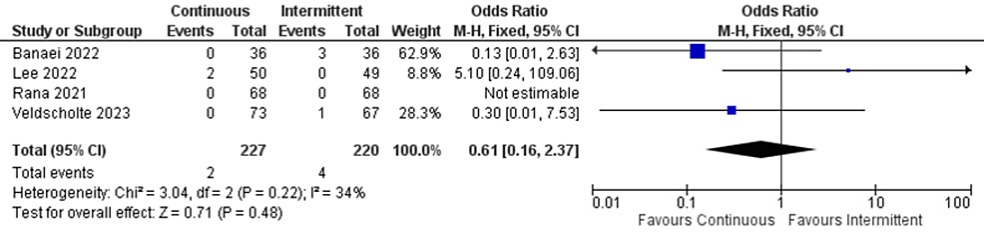
Forest plot of aspiration. CI: confidence interval; M-H: Mantel-Haenszel

Aspiration: Only four studies reported data about aspiration. Similarly, there was no significant difference between continuous and intermittent enteral feeding (OR: 0.61; 95% CI: 0.16 to 2.73; p=0.48; I^2^=34% {moderate heterogeneity}) (Figure 12) [4,18,19,23].

**Figure 13:**
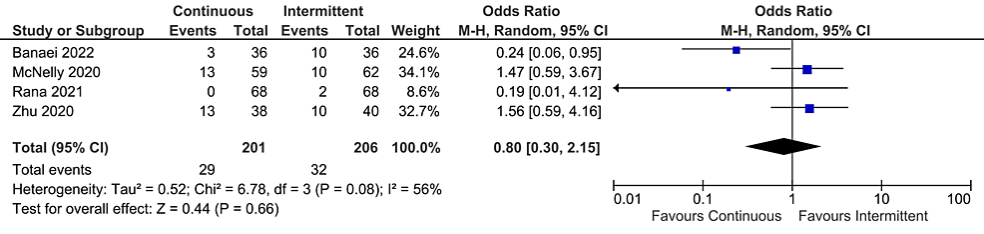
Forest plot of gastric residual volume (GRV). CI: confidence interval; M-H: Mantel-Haenszel

Gastric residual volume (GRV): Data from four studies was analyzed for GRV. Similarly, there was no significant difference between continuous and intermittent enteral feeding (OR: 0.80; 95% CI: 0.30 to 2.15; p=0.66; I^2^=56% {high heterogeneity}) (Figure 13) [4,12,22,23].

Discussion

Currently, there are only a limited number of clinical trials published to assess the difference between intermittent and continuous enteral feeding methods. This systematic review and meta-analysis aimed to compare the outcomes of bolus versus continuous enteral feeding in critically ill patients. In the present systemic review and meta-analysis, there was no significant difference in any outcome in both bolus and continuous enteral feeding groups. Despite this fact, both ICU mortality and hospital mortality were close to achieving statistical significance (p=0.07 and p=0.06, respectively). Both these outcomes favored continuous feeding approaches. However, more studies are required to prove this association. Furthermore, the total number of studies and patients is small, therefore, it is not possible to draw specific conclusions. The included RCTs encompassed a diverse population of critically ill patients, including adults and children, with varying conditions such as trauma, mechanical ventilation, and hemorrhagic strokes.

Our findings are supported by a previous meta-analysis that revealed that there was no difference in continuous and intermittent feeding. However, they reported that continuous enteral feeding reduced the diarrhea (risk ratio=0.42) [11]. Similarly, a recent meta-analysis by Heffernan et al. reported that there was an increased risk of constipation in the continuous feeding group compared to the bolus feeding approach. However, they also found an insignificant difference in other outcomes [8]. This pattern can be explained by the fact that gut motility primarily depends on stimulation from intraluminal contents. This is evident that intermittent feeding includes delivering larger intraluminal contents compared to continuous feeding where smaller amounts are given at a slow rate [24]. Furthermore, continuous feeding often requires prolonged bed rest to ensure non-interruptible enteral nutrition. Prolonged bed rest can serve as a contributor to constipation as well [25]. Previous research has shown that both continuous and bolus-feeding practice has their pros and cons; however, they are both effective and practical approaches [26]. There was no statistically significant difference in metabolite patterns between bolus and continuous feeding in the present meta-analysis. Wilkinson et al., in their RCT, investigated 594 samples and 87 metabolites. Their findings showed no significant differences between feeding arms or over time and no correlation was found with quadriceps muscle mass changes [17]. Similarly, McNelly et al. reported no significant difference in muscle loss between bolus and continuous feeding, indicating that intermittent nutritional feeding may be as effective as continuous feeding in preserving muscle mass. However, they identified that intermittently fed patients achieved higher protein (p<0.001) and energy (p=0.001) targets [12].

Our findings are not consistent with the meta-analysis of Ma et al. that included 14 clinical trials comprising 1025 critically ill patients [27]. They found that intermittent feeding was associated with an increased risk of feeding intolerance, GRV, and aspiration compared to continuous feeding. However, most of the included studies in their meta-analysis had a high risk of bias. Generally, critically ill patients are more likely to develop feeding intolerance. However, no study included in this meta-analysis found significant differences in these outcomes. Veldscholte et al. focused on critically ill children and concluded that condensed feeding periods during the day did not lead to increased feeding intolerance [18]. However, intermittent feeding with an overnight fast resulted in lower calorie intake. Lee et al. reported that continuous feeding achieved targeted nutrition more significantly than bolus feeding, although clinical outcomes, including ICU length of stay, gastrointestinal intolerance, mortality, and organ support, did not differ significantly [19]. According to the European Society for Clinical Nutrition and Metabolism (ESPEN) guidelines, it is recommended that critically ill patients requiring nutritional support should be offered enteral feeding within 48 hours [11]. These guidelines recommended continuous feeding rather than intermittent; however, we were unable to find any significant difference between continuous and intermittent feeding approaches. The quality of evidence included in our meta-analysis was high.

There are some limitations of the meta-analysis as well which should be kept in mind while interpreting the findings. First, the majority of the studies had a small sample size. Second, not all studies reported all the outcomes assessed in this meta-analysis. Therefore, the findings might not be supported by all studies. Third, in the majority of the studies, physicians or participants were not blinded, leading to high-performance bias. Fourth, we limited the duration of the search to five years which could impact the generalizability of the findings. The addition of prior studies might have impacted the overall findings of the meta-analysis. Apart from these, we only included studies that were published in English. There is also no report on the type of patients included in the studies.

## Conclusions

In conclusion, the systematic review and meta-analysis indicate that bolus and continuous enteral feeding methods have comparable outcomes in critically ill patients. The choice between these methods may depend on patient-specific factors, preferences, and clinical context. The findings emphasize the importance of tailoring enteral feeding strategies to individual patient needs and highlight the need for further research in this area. The high-quality assessment of the included studies adds credibility to the overall findings of this systematic review.

## Appendices

**Table 2:** Terms and strategy used for literature search. MeSH: Medical Subject Headings; Ti/Ab: title/abstract; ICU: intensive care unit

(1) Intensive care units (MeSH)	(10) Bolus
(2) Critical care (MeSH)	(11) Sequential
(3) Critical illness (MeSH)	(12) OR/8-11
(4) Critically ill (Ti/Ab)	(13) Feed
(5) ICU (Ti/Ab)	(14) Feeding
(6) Intensive care (Ti/Ab)	(15) Enteral
(7) OR/1-6	(16) Enteral feeding
(8) Continuous	(17) OR/13-16
(9) Intermittent	(18) 7 AND 12 AND 17
